# Early myeloid-derived suppressor cells accelerate epithelial-mesenchymal transition by downregulating ARID1A in luminal A breast cancer

**DOI:** 10.3389/fbioe.2022.973731

**Published:** 2022-10-18

**Authors:** Guidong Chen, Xingchen Li, Chenyan Ji, Pengpeng Liu, Li Zhou, Dechen Xu, Dong Wang, Jie Li, Jinpu Yu

**Affiliations:** ^1^ Cancer Molecular Diagnostics Core, Tianjin Medical University Cancer Institute and Hospital, National Clinical Research Center for Cancer, Tianjin’s Clinical Research Center for Cancer, Tianjin, China; ^2^ Key Laboratory of Breast Cancer Prevention and Therapy, Tianjin Medical University, Ministry of Education, Tianjin, China; ^3^ Key Laboratory of Cancer Immunology and Biotherapy, Tianjin, China; ^4^ School of Computer Science and Technology, Harbin Institute of Technology, Harbin, China

**Keywords:** breast cancer, early-stage myeloid-derived suppressor cells, ARID1A, epithelial-mesenchymal transition, prognostic factor

## Abstract

Early myeloid-derived suppressor cells (eMDSCs) are a newly characterized subclass of MDSCs, which exhibit more potent immunosuppressive capacity than classical MDSCs. Previously, we found high eMDSCs infiltration was correlated with poor prognosis of breast cancer, though the regulatory mechanisms have not been fully understood. Here, we constructed a 21-gene signature to evaluate the status of eMDSCs infiltration within breast cancer tissues and found that highly infiltrated eMDSCs affected the prognosis of breast cancer patients, especially in luminal A subtype. We also found that eMDSCs promoted epithelial-mesenchymal transition (EMT) and accelerated cell migration and invasion *in vitro*. Meanwhile, eMDSCs significantly downregulated ARID1A expression in luminal A breast cancer, which was closely associated with EMT and was an important prognostic factor in breast cancer patients. Moreover, significant changes of EMT-related genes were detected in luminal A breast cancer cells after co-cultured with eMDSCs or ARID1A knock-down and overexpression of ARID1A significantly reversed this procedure. These results implied that eMDSCs might suppress the ARID1A expression to promote EMT in luminal A breast cancer cells, which might provide a new light on developing novel treatment regimens for relapsed luminal A breast cancer after conventional therapies.

## Introduction

Breast cancer (BC) is one of the most common malignant tumors in women and can be divided into four subtypes: luminal A, luminal B, HER2^+^, and basal-like (Onitilo et al., 2009). Approximately 80% of breast cancer patients present as ER positive (luminal A and luminal B), and estrogen stimulates the development of ER^+^ breast cancers by increasing breast cancer cell survival and proliferation (Thomas and Gustafsson, 2011; Clarke et al., 2015; Thomas and Gustafsson, 2015). However, clinical investigations have shown that although surgery, chemotherapy, radiotherapy and anti-estrogen therapy are effective in reducing mortality in breast cancer patients, tumor recurrence and metastasis are the leading cause of mortality in these individuals (Carroll, 2016; Razavi et al., 2018). Accumulating evidence indicates that epithelial-mesenchymal transition (EMT), a process of initial step for tumor metastasis, gives cells the extra plasticity needed for invasion and metastasis to distant organs (Mittal, 2018; Dongre and Weinberg, 2019; Pastushenko and Blanpain, 2019). Furthermore, EMT endows tumor cells with cancer stem cell-like characteristics, making them resistant to therapies and prone to recurrence following therapy (Shibue and Weinberg, 2017; Erin et al., 2020). Therefore, a deeper knowledge of tumor growth and metastasis molecular pathways is essential to assist the creation of more accurate prognostic indicators as well as efficient treatment options.

Crosstalk between cancer cells and the surrounding microenvironment appears to play an essential role in the recurrence and metastasis of many malignancies, according to growing research (Wang et al., 2018; Hinshaw and Shevde, 2019; Lan et al., 2019; Zavros and Merchant, 2022). Myeloid-derived suppressor cells (MDSCs) are a prominent component of the tumor microenvironment, and their defining characteristic is their powerful immune suppressive action (Ma et al., 2011; Hegde et al., 2021). As a diverse group of immature myeloid cells, MDSCs have strong protumoral capacity by suppressing innate and adaptive immunity, promoting angiogenesis and stimulating tumor cell invasion and are further divided into three subsets: M-MDSCs (monocytic myeloid-derived suppressor cells), PMN-MDSCs (polymorphonuclear MDSCs) and eMDSCs (early-stage MDSCs) (Bronte et al., 2016). eMDSCs are a newly defined subset of MDSCs, whose phenotype in breast cancer were defined as Lin^−^HLA-DR^-^CD45^+^CD33^+^CD13^+^CD14^−^CD15^−^ in humans and CD11b^+^Gr-1^-^F4/80^-^MHC-II^-^ in mice (Zhou et al., 2010; Yu et al., 2013; Yu et al., 2014; Jiang et al., 2017; Zhang et al., 2018). Furthermore, eMDSCs are the most predominant subpopulation of MDSCs in breast cancer and exhibit enhanced immunosuppressive capacity in the tumor microenvironment compared to classical MDSCs (Yu et al., 2013; Yu et al., 2014; Jiang et al., 2017).

Although the concept of immunosuppressive MDSCs is well established, it could also contribute to tumor development through a variety of non-immunological pathways, such as promoting angiogenesis, enhancing tumor cell stemness, and maintaining pre-metastatic niche (Safarzadeh et al., 2018; Wang et al., 2019; Tang et al., 2021). Previously, we found high eMDSCs infiltration was correlated with poor prognosis of breast cancer (Jiang et al., 2017). However, whether eMDSCs promote tumor invasion and metastasis via EMT-related non-immunological pathways and then lead to tumor recurrence and poor prognosis of breast cancer is not fully understood.

Therefore, in this study we established a 21-gene signature to predict the infiltration of eMDSCs within breast cancer tissues which is a valuable predictive biomarker for luminal A breast cancer patients. Moreover, we found that eMDSCs suppress the ARID1A expression to promote EMT in luminal A breast cancer cells, which might provide a new light on developing novel treatment regimens for relapsed luminal A breast cancer after conventional therapies.

## Materials and methods

### Clinical samples and healthy donors

We obtained 280 primary breast cancer tissue samples from two cohorts (Shanghai Outdo Biotech Co., Ltd.), whose tumor tissues were constructed into tissue arrays following surgical resection between January 2001 and December 2008. All patients were females, with a median age of 51 years (range: 29–83 years) in cohort 1 and 57 years (range: 37–88 years) in cohort 2. Infiltrating mammary-ductal cancer accounted for 93.6% (131/140) in cohort 1 and 97.8% (137/140) in cohort 2. The cohort 1 included 11 patients with clinical stage I, 80 patients with clinical stage II, and 47 patients with clinical stage III cancer, respectively. The cohort 2 included 3 patients with clinical stage I, 62 patients with clinical stage II, and 65 patients with clinical stage III cancer, respectively. The Cohort 1 included 79, 17, and 26 patients with luminal, HER2 overexpression, and TNBC cancer subtypes, respectively (Table 1). The Cohort 2 included 62, 43, and 35 patients with luminal, HER2 overexpression, and TNBC cancer subtypes, respectively (Table 1). Three individuals of the cohort 1 were dropped from the research due to non-cancer related deaths.

**TABLE 1:** Baseline of all patients.

Baseline	Cohort 1	Cohort 2
Total	140	140
**Age**		
≤50 years	67	39
>50 years	73	101
**Tumor size**		
≤2 cm	30	33
>2 cm	110	107
**Lymph nodes**		
Negative	84	66
Positive	52	69
**Stage**		
I	11	3
II	80	60
III	47	64
**Subtype**		
Luminal	79	62
HER2 overexpressing	17	43
TNBC	26	35

Fourteen fresh primary breast cancer tissue samples were acquired to validate the efficiency of the 21-gene signature in predicting eMDSCs infiltration *in situ.* Peripheral blood (PB) samples were taken from healthy volunteers in order to isolate CD33^+^ myeloid progenitors.

### Cell line and cell culture

ATCC provided the breast cancer cell lines MCF-7, T47D, and EO771. MCF-7, and EO771 cell lines were grown in full DMEM (Gibco, Grand Island, NY, United States) with 10% FBS in a 5% CO_2_ incubator at 37°C. T47D cells were grown in full RPMI 1640 media (Gibco, Grand Island, NY, United States) with 10% fetal bovine serum in a 5% CO_2_ incubator at 37°C.

### Animal experiments

The mice (Beijing Vital River Facility Animal Technology, Beijing, China) were kept at the Tianjin Medical University Cancer Institute and Hospital’s Specific pathogen free (SPF) animal laboratory. For constructing abundant tumor-infiltrating eMDSCs mice model, conditional targeting was performed as previously described (Yasukawa et al., 2003). An ES targeting technique was utilized to establish conditional SOCS3 knockout C57BL/6 mice. A targeting vector with recombination sites and selection markers was constructed. F1 SOCS3^fl/+^ mice were constructed by Cyagen Biosciences Inc (Guangzhou, China). F1 SOCS3^fl/+^ mice were intercrossed to generate SOCS3^fl/fl^ mice. LysM-cre mice (cDNA for cre was inserted into the lysozyme M gene) were crossed with SOCS3^fl/fl^ mice. These mice were further intercrossed to generate LysM-cre SOCS3^fl/fl^ (SOCS3^KO^) mice. In addition, SOCS3^fl/fl^ mice were used as control group.

For *in vivo* growth assay, EO771 cells (1×10^6^ cells) were injected into the mammary fat pads of 6-week-old female SOCS3^KO^ and SOCS3^fl/fl^ mice. Every 3 days, the tumor volume and body weight were measured. After 15 days, all animals were euthanized under anesthesia by cervical dislocation, and the tumors were collected for future research.

### Isolation of mice early myeloid-derived suppressor cells

The bone marrow or tumor tissues of SOCS3^KO^ and SOCS3^fl/fl^ mice were used to isolate murine CD11b^+^Gr-1^-^ eMDSCs as described previously (Zhang et al., 2018). Bone marrow or tumor tissues were prepared into single-cell suspensions. CD11b^+^Gr-1^−^ eMDSCs were isolated following erythrocytolysis using anti-mouse Gr-1 coupled with biotin and anti-biotin microbeads (Miltenyi Biotec, Germany). The BD FACSAria™ II cell sorter (BD Biosciences, San Jose, CA, United States) was used to isolate CD11b^+^Gr-1^−^F4/80^−^MHCII^−^ eMDSCs. Flow cytometry and trypan blue staining were used to examine the vitality and purity of the retrieved cells.

### Detection of primary early myeloid-derived suppressor cells *in situ* and induction of human early myeloid-derived suppressor cells *in vitro*

The extent of eMDSCs infiltration in the 14 fresh primary breast cancer tissue samples and 280 primary breast cancer tissue samples from two cohorts were examined using immunohistochemistry (IHC) assay and multispectral IF staining to detect the expression of CD33 protein as described previsouly (Yu et al., 2013, 2014; Jiang et al., 2017). The median number of positive staining was applied as the threshold of eMDSCs. Therefore, the samples with lower amount than the threshold were regarded as low infiltration of eMDSCs, while the samples with higher amount than the threshold were regarded as high infiltration of eMDSCs.

CD33^+^ cells were extracted from healthy PBMCs using human CD33 MicroBeads (130-045-501; Miltenyi Biotec, Germany) and co-cultured with MDA-MB-231 cells to create human eMDSCs, as previously described (Jiang et al., 2017).

### Immunosuppressive capacity of early myeloid-derived suppressor cells

T cells were extracted from normal C57BL/6 mice using the Pan T Cell Isolation Kit (Miltenyi Biotec) and co-cultured in 24-well plates with either BM- or tumor-derived eMDSCs from tumor-bearing mice at a 1:3 ratio in RPMI-1640 media supplemented with 10% FBS. To excite T cells *in vitro*, anti-CD3/CD28 beads (20 μL/10^6^ cells; Gibco) were used. T cell proliferation and apoptosis were investigated using the BrdU and Annexin V labeling assays described before (Yu et al., 2013).

### Tumor invasion induced by early myeloid-derived suppressor cells

The isolated human and mice eMDSCs were placed into a 0.4 μm co-incubation chamber and cocultured with T47D, MCF-7, or EO771 cell at 1:3 ratio. After 2 days, the cells were collected for Transwell assay and EMT markers detection.

### Flow cytometry analysis

Human eMDSCs were labeled using CD45, CD13, CD33, CD14, and CD15 antibodies (BD Biosciences, United States). Mouse eMDSCs were detected using CD45, CD11b, Gr-1, F4/80, and MHC-II antibodies (Biolegend, United States). A BD FACS Canto™ II flow cytometer was used for the study (BD Biosciences, United States). Our prior paper detailed the gating technique (Jiang et al., 2017).

### RNA-seq analysis and construction of the 21-genes signature prediction model

Novogene Co., Ltd. was tasked with library preparation and sequencing. To screen for potential candidates that might predict the infiltration of eMDSCs within breast cancer tissues, 424 differentially expressed genes were selected from the expression profiles of eMDSCs^SOCS3KO^, CD11b^+^GR-1^+^, and eMDSCs^fl/fl^ cells by the Z-Score-like method as previously described (Li et al., 2010). Then, 50 genes that can separate the three groups well are selected and mapped to human genes to obtain 42 genes. We validated the 42 genes in human PBMC-derived eMDSCs using qRT-PCR. Finally, 21 genes with comparable transcriptional patterns in humans and mice were selected to construct the genetic prediction model.

### siRNA interference and gene transfection

pcDNA3.1-ARID1A overexpression vector or pcDNA3.1 empty vector and/or siRNAs against ARID1A or negative control (Shanghai HanBio Co., Ltd.) were delivered using Lipofectamine^®^ 2000 according to the manufacturer’s procedure (Invitrogen; Thermo Fisher Scientific, Inc.). After 6 h, the reduced-serum media was replaced with full medium. The cells were extracted after 48 h to be used in following studies.

### Immunohistochemistry

Following surgical excision, fresh tissues were promptly fixed with formalin; tissues were then embedded in paraffin and cut into 4-µm serial sections. Deparaffinization and rehydration of breast cancer tissue samples followed. After inhibiting endogenous peroxidase with 3% H_2_O_2_, citrate-based antigen retrieval was carried out. Primary antibodies were incubated overnight at 4°C on the samples. Following an incubation with the secondary antibody, a DAB Substrate kit was used to stain the sections according to the manufacturer’s instructions. Five representative high-power fields (400 magnification) for each tissue section were selected for histology evaluation as previously described (Yu et al., 2013). The average amount of positive staining cells among 5 fields was calculated for each sample.

### Multispectral IF staining

The breast cancer tissue samples were embedded in paraffin, deparaffinized and rehydrated, incubated with antibodies and imaged using the multiplex method as previously described (Ning et al., 2021). Five fields from each slide were imaged and scored. The average amount of positive staining cells among 5 fields was calculated for each sample.

### Transwell assay

Before plating in the upper chamber, T47D, MCF-7, and EO771 cell lines were collected and resuspended in serum-free DMEM. 1640 or DMEM supplemented with 10% FBS was used to fill the lower chamber of the Transwell. The cell suspension was placed to the membrane, either with or without Mtrigel adhesive, and incubated for 48 h at 37°C. All of the trials were carried out three times.

### qRT-PCR analysis

qRT-PCR was used to examine the mRNA expression of ARID1A, Vimentin and E-Cadherin in breast cancer cell lines. Table 2 displays the primers for ARID1A, Vimentin, and E-Cadherin. As an internal control, β-actin was employed. Each sample was calculated using the formula: 2^−ΔCt^ (ΔCt = Ct_gene_–Ct_β-actin_). Every test was carried out at least three times.

**TABLE 2:** The RT-PCR primers of interested genes.

Genes	Primer sequences
β-actin (mouse)	Up GTG​CTA​TGT​TGC​TCT​AGA​CTT​CG
	Down ATG​CCA​CAG​GAT​TCC​ATA​CC
β-actin (human)	Up TGG​CAC​CCA​GCA​CAA​TGA​A
	Down CTA​AGT​CAT​AGT​CCG​CCT​AGA​AGC​A
ARID1A (mouse)	Up AGT​CCC​AGC​AAA​CTG​CCT​AT
	Down TTC​TTG​CCC​TCC​CTT​ACT​GG
ARID1A (human)	Up GGG​CGT​AAT​GAC​ATG​ACC​TAT​A
	Down CAT​TTC​ATC​TGT​TCG​GTT​CAC​G
Vimentin (mouse)	Up ACC​CTG​CAG​TCA​TTC​AGA​CA
	Down AGT​GAG​GTC​AGG​CTT​GGA​AA
Vimentin (human)	Up CCC​TCA​CCT​GTG​AAG​TGG​AT
	Down TGA​CGA​GCC​ATT​TCC​TCC​TT
E-Cadherin (mouse)	Up CAG​GTC​TCC​TCA​TGG​CTT​TGC
	Down CTT​CCG​AAA​AGA​AGG​CTG​TCC
E-Cadherin (human)	Up CTTCGGAGGAGAGCGGTG
	Down CTAGTCGTCCTCGCCGCC

### Western blot analysis

SDS-PAGE was used to separate cell lysates, which were then transferred to polyvinylidene difluoride membranes and incubated with rabbit antibody ARID1A (abcam, ab182560, United States), E-Cadherin (CST, 3195, United States), Vementin (CST, 5741, United States), and SNAIL (CST, 3879, United States) over night at 4°C. Following an incubation with the secondary antibody, the chemiluminescent substrate kit was used to visualize the protein bands. The relative densities of target protein were determined by comparing with GAPDH.

### Immunofluorescence

Breast cancer cells were placed on cover glasses and allowed to cling to the surface. With 0.1 percent Triton X-100, the cells were fixed and permeabilized. They were then blocked with 5% BSA and treated at 4°C overnight with primary antibodies. Following an incubation with the secondary antibody for 1 h, the Iamger.Z2 (Zeiss, Oberkochen, Germany) was used to visualize the protein.

### Statistical analysis

GraphPad Prism 8.0.2 software were used for statistical analyses. The Kaplan–Meier technique was employed to calculate the cumulative survival probability, and the log-rank test was utilized to compare the OS of each patient subgroup. All experiments were independently performed at least three times. The values are presented as mean ± standard deviation (SD). *p* < 0.05 was chosen as the level of statistical significance.

## Results

### 21-genes signature predicting highly early myeloid-derived suppressor cells infiltration *in situ* are significantly correlated with poor prognosis in breast cancer patients

Suppressor of cytokine signaling-3 (SOCS3), a known feedback inhibitor of the Janus kinase-signal transducer and activator of transcription 3 (JAK/STAT3) signaling pathway, is involved in the differentiation of eMDSCs (Jiang et al., 2017; Zhang et al., 2018). In this study, we successfully constructed a conditionally SOCS3 gene knockout mouse model in the myeloid lineage by using the murine Cre-loxP system (SOCS3^KO^) and detected a significant increase in the amount of CD11b^+^Gr-1^−^ eMDSCs both in bone marrow (eMDSCs^SOCS3KO^) and tumor tissues (eMDSCs^Tumor^) of tumor-bearing SOCS3^KO^ mice. To analyze their immunosuppressive capacity, naïve T cells from wild-type C57BL/6 mice were co-cultured with eMDSCs^SOCS3KO^ or eMDSCs^Tumor^. We found that eMDSCs^SOCS3KO^ and eMDSCs^Tumor^ significantly inhibited T cell proliferation (33.0 ± 3.4% vs. 18.1 ± 4.2% vs. 19.8 ± 3.6%, *p* = 0.0086 and 0.0097; Supplementary Figure S1A) and promoted T cell apoptosis (3.5 ± 0.9% vs. 17.3 ± 1.4% vs. 15.4 ± 1.5%, *p* = 0.0001 and 0.0003, Supplementary Figure S1B). This indicated that eMDSCs exerted exceptional T cell immunosuppressive ability *in vitro* and *vivo*.

**FIGURE 1:**
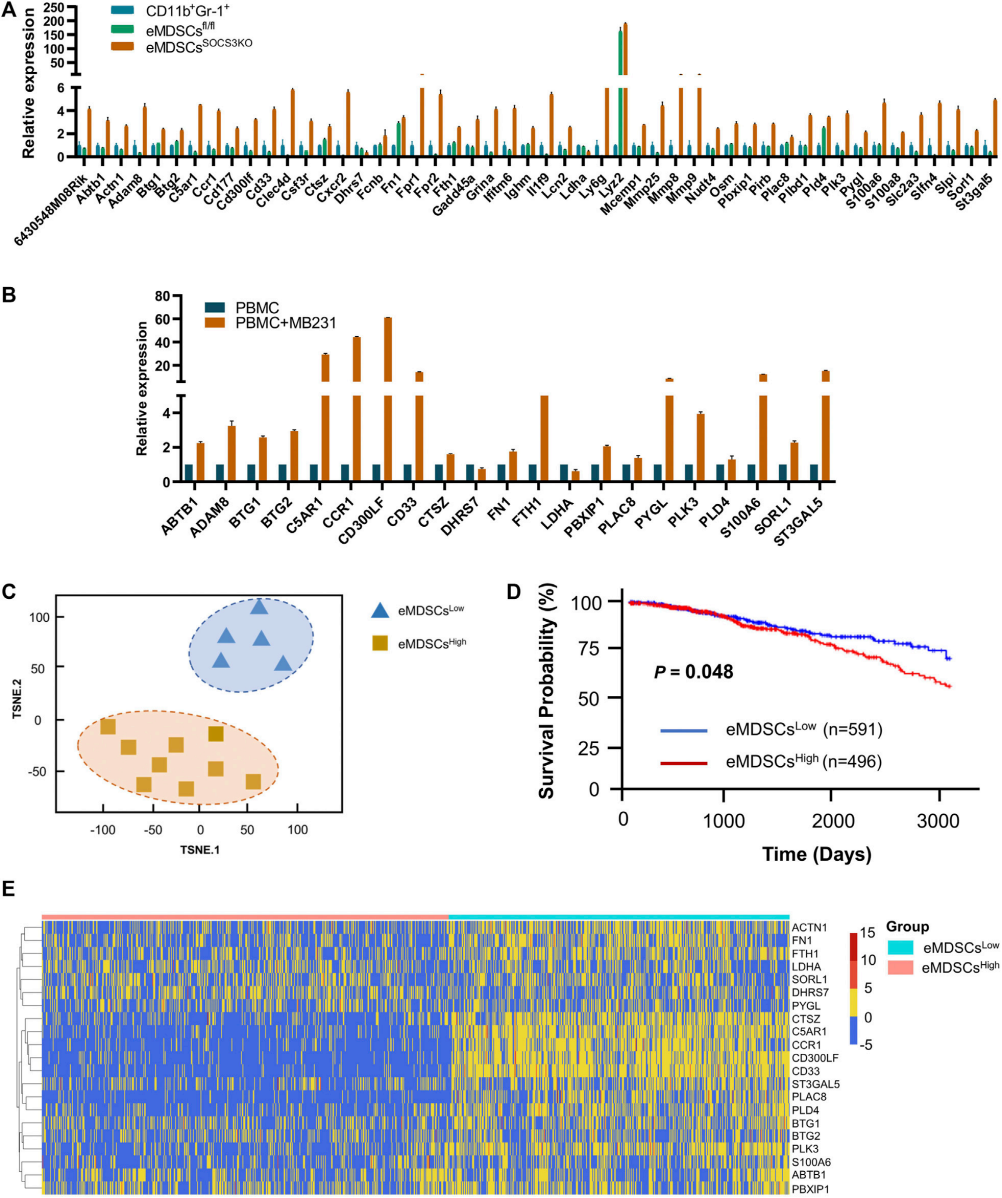
The 21-genes signature is able to predict the infiltration of eMDSCs *in situ*.**(A)** The relative expression of 50 genes that can separate the three groups of eMDSCs^SOCS3KO^, CD11b^+^Gr-1^+^ and eMDSCs^fl/fl^ cells. **(B)** The relative expression of 21 genes in human PBMC-derived eMDSCs was confirmed by RT-qPCR. The transcriptional patterns of 21 genes are comparable both in humans and mice. CD33^+^ myeloid progenitors isolated from healthy PBMCs were co-cultured with MDA-MB-231 breast cancer cells to induce eMDSCs. **(C)** T-SNE analysis was used to visually display the classification results and the consistency between the 21-genes signature prediction and immunohistochemical observation was as high as 85.7% (12/14). **(D)** Kaplan-Meier survival analysis of differences between eMDSCs^high^ and eMDSCs^low^ groups in TCGA breast cancer cohort showed that the OS was longer in the eMDSCs^low^ group than the eMDSCs^high^ group. **(E)** Heatmap of the 21 genes expression profiles in the TCGA breast cancer cohort.

Using this mouse model, we obtained a sufficient number of eMDSCs to perform RNA sequencing and compared the genomic expression profile of eMDSCs^SOCS3KO^ (CD11b^+^Gr-1^-^) cells with classical MDSCs (CD11b^+^Gr-1^+^) cells or with normal myeloid precursors eMDSCs^fl/fl^. The differentially expressed genes in eMDSCs^SOCS3KO^ compared to those in CD11b^+^Gr-1^+^, and eMDSCs^fl/fl^ were filtered to screen for potential candidates that might predict the infiltration of eMDSCs within breast cancer tissues as previously described (Li et al., 2010). 50 genes that can separate the three groups well are selected and validated them in human PBMC-derived eMDSCs using qRT-PCR (Figures 1A,B). Finally, 21-genes signature, including ABTB1, ACTN1, BTG1, BTG2, C5AR1, CCR1, CD300LF, CD33, CTSZ, DHRS7, FN1, FTH1, LDHA, PBXIP1, PLAC8, PYGL, PLK3, PLD4, S100A6, SORL1, and ST3GAL5 were selected to construct a genetic prediction model.

In order to verify the efficacy of 21-genes signature to predict the infiltration of eMDSCs *in situ*, 14 primary breast cancer tissue samples were collected and eMDSCs in breast cancer tissues were detected by IHC staining. Breast cancer samples were separated into two groups based on the median number of CD33^+^ eMDSCs infiltrated locally: lowly infiltrated eMDSCs (eMDSCs^low^, *n* = 7) and highly infiltrated eMDSCs (eMDSCs^high^, *n* = 7). The two groups of breast cancer tissue samples were subjected to RNA-seq analysis and were further analyzed using the 21-genes signature. As expected, the consistency between the 21-genes signature and IHC assay was as high as 85.7% (12/14), implying that the 21-genes signature accurately predicted the infiltration status of eMDSCs *in situ* (Figure 1C).

Therefore, we enrolled a total of 1087 cases of breast cancer samples in TCGA dataset and divided them into eMDSCs^high^ (*n* = 496) and eMDSCs^low^ (*n* = 591) groups using the 21-genes signature (Figure 1D). The heatmap of the 21 genes expression profiles in TCGA breast cancer cohort is presented in Figure 1E. Of these 21 genes, high expression of ABTB1, ACTN1, BTG1, BTG2, C5AR1, CCR1, CD300LF, CD33, CTSZ, FN1, FTH1, PBXIP1, PLAC8, PYGL, PLK3, PLD4, S100A6, SORL1, and ST3GAL5 indicated to be associated with highly infiltrated eMDSCs, while high expression of DHRS7 and LDHA was associated with lowly infiltrated eMDSCs. The Kaplan-Meier survival analysis indicated that the median survival of eMDSCs^high^ breast cancer patients was significantly shorter than eMDSCs^low^ breast cancer patients, consistent results were validated in 3 more breast cancer datasets, including GSE3143 (*n* = 158), GSE48408 (*n* = 164), and GSE9893 (*n* = 155) (Figure 1D; Supplementary Figure S2).

**FIGURE 2:**
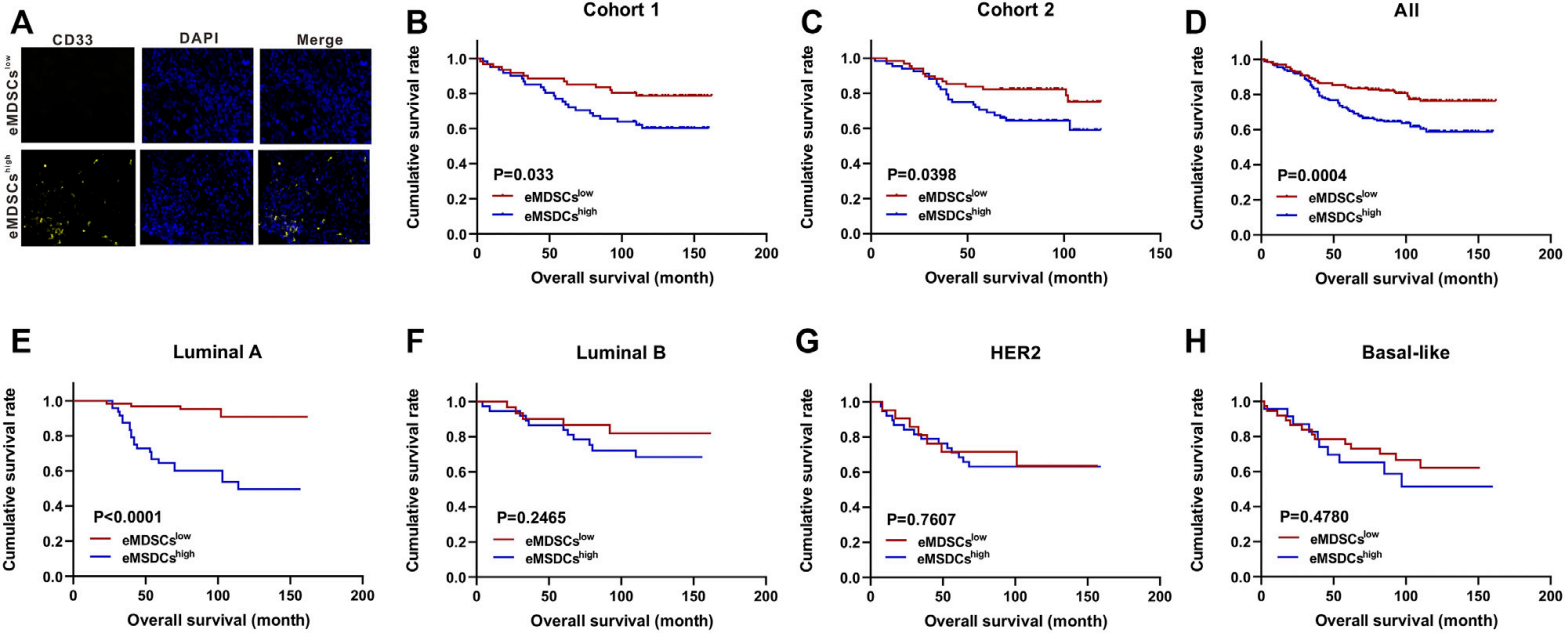
Highly-infiltrated eMDSCs are significantly correlated with poor prognosis in breast cancer patients **(A)** Multispectral IF staining analysis showed the infiltration of CD33^+^ cells in tumor sections from 280 cases of breast cancer patients. **(B–H)** Kaplan-Meier survival analysis of overall survival between eMDSCs^high^ and eMDSCs^low^ groups among 4 different subtypes of 280 cases of breast cancer patients.

Multispectral IF staining was performed to detect the expression of CD33 protein in 280 primary breast cancer patients from two cohorts. According to the median number of CD33^+^ eMDSCs that infiltrated locally, breast cancer patients were categorized into eMDSCs^high^ and eMDSCs^low^ groups (Figure 2A). We found that the OS of eMDSCs^high^ patients was significantly shorter than those of eMDSCs^low^ patients in the cohort 1 (*p* = 0.033, Figure 2B), cohort 2(*p* = 0.0398, Figure 2C) and the combined cohorts 1 and 2 (*p* = 0.0004, Figure 2D). Furthermore, when we compared the OS among four different molecular subtypes of all 280 cases of breast cancer patients, highly infiltrated eMDSCs strongly correlated with poor patient outcome in luminal A subtype (*p* < 0.0001, Figure 2E), rather than in other subtypes such as luminal B, HER2 overexpression and Basal-like (*p* = 0.2465, *p* = 0.7607, *p* = 0.4780, Figures 2F–H).

### Early myeloid-derived suppressor cells promote migration and invasion of luminal A breast cancer cells *via* stimulating epithelial-mesenchymal transition

**FIGURE 3:**
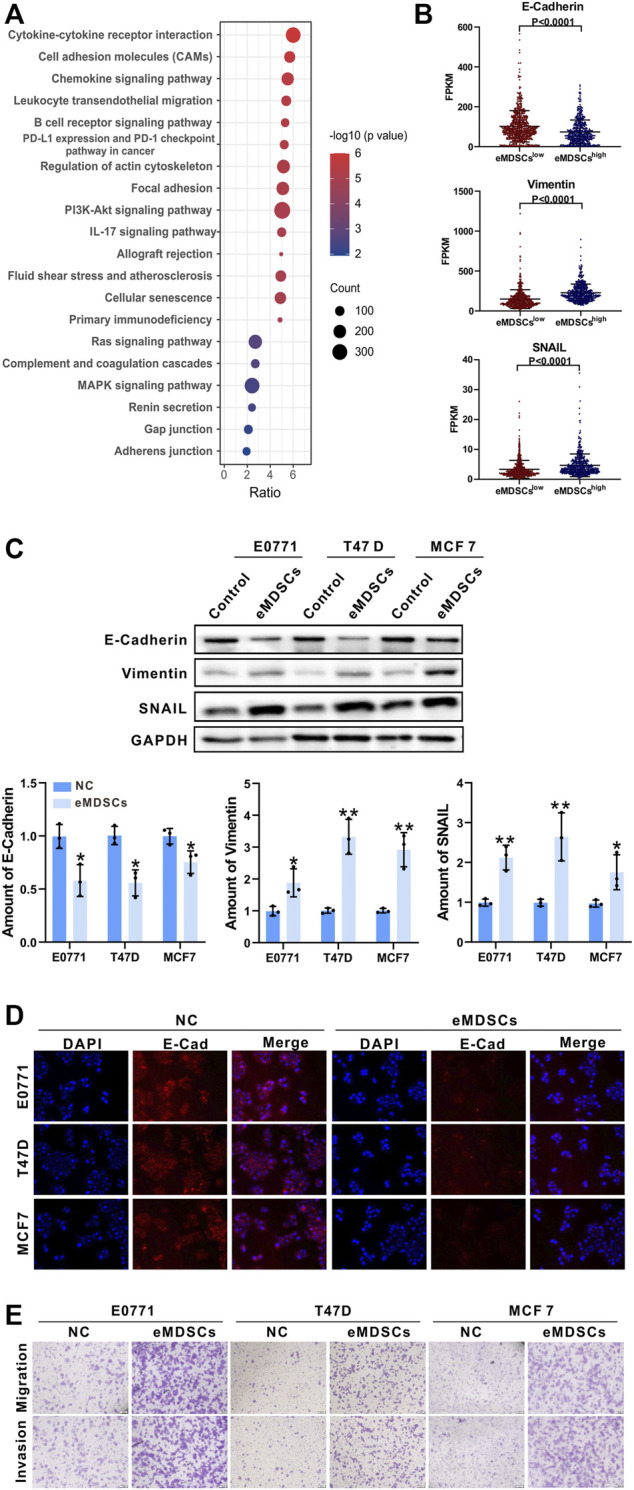
eMDSCs promote migration and invasion of luminal A breast cancer cells *via* stimulating EMT **(A)** KEGG enrichment analysis of the differential expressed genes between eMDSCs^high^ and eMDSCs^low^ groups divided by the 21-genes signature in TCGA breast cancer cohort. **(B)** Distribution of FPKM values for TCGA breast cancer samples between eMDSCs^high^ and eMDSCs^low^ groups for the three EMT-related genes. **(C)** Immunoblotting was used to assess the proteins of E-Cadherin, Vimentin, and SNAIL in the indicated breast cancer cells. **(D)** Immunofluorescence was used to examine the levels of E-Cadherin in EO771, T47D, and MCF7 cells cocultured with eMDSCs. **(E)** Transwell assays revealed that eMDSCs dramatically improved the migratory and invasion capacities of EO771, T47D, and MCF7 cells.

We performed gene expression and pathway enrichment analysis between eMDSCs^high^ and eMDSCs^low^ groups in TCGA database using the 21-genes signature, and found that the cell adhesion molecules, focal adhesion, gap junction and adherens junction were significantly enriched as suggested by KEGG analysis (Figure 3A). It has been observed that cells communicate with one another through subapical tight junctions, adherens junctions and desmosomes at lateral surfaces, and scattered gap junctions at lateral surfaces, all of which are required for epithelial integrity (Huang et al., 2012). When EMT begins, these junctions are dismantled, and the junction proteins are relocalized and/or destroyed. These data suggested that eMDSCs may promote breast cancer metastasis *via* accelerating EMT.

The loss of E-Cadherin protein was thought to be the start of EMT, whereas the gain of Vimentin and SNAIL gave tumor cells increased migratory ability (Lamouille et al., 2014). TCGA database analysis showed that E-Cadherin was reduced and Vimentin and SNAIL were elevated in eMDSCs^high^ breast cancer patients relative to eMDSCs^low^ group (Figure 3B). To verify the above results, we co-cultured eMDSCs with 3 luminal A breast cancer cell lines (mouse mammary tumor cell line EO771, human mammary tumor cell line MCF7 and T47D) *in vitro*. Western blot and immunofluorescence analyses were also done to identify the expression levels of E-Cadherin, Vimentin and SNAIL in luminal A breast cancer cell lines after being co-cultured with eMDSCs. In line with the results shown above, eMDSCs significantly suppressed the expression of E-Cadherin and enhanced the expression of Vimentin and SNAIL (Figures 3C,D). Moreover, the migration and invasion of EO771, MCF7, and T47D cells were greatly accelerated following co-culture with eMDSCs (Figure 3E). Taken together, these results suggest that eMDSCs promoted migration and invasion of luminal A breast cancer cells *via* stimulating EMT.

### Early myeloid-derived suppressor cells downregulate the expression of ARID1A in luminal A breast cancer cells

**FIGURE 4:**
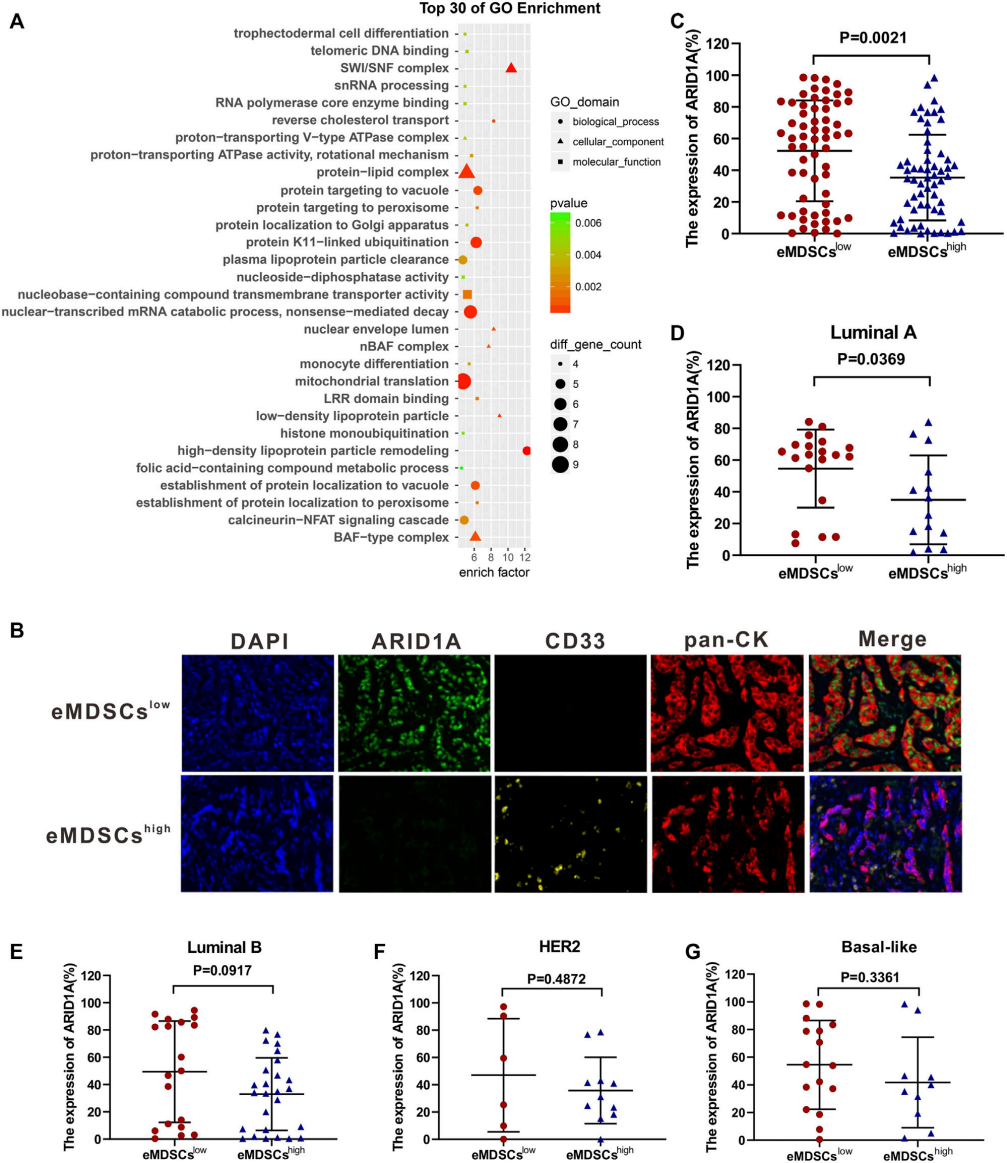
eMDSCs downregulate the expression of ARID1A in luminal A breast cancer cells **(A)** Gene ontology enrichment analysis of the strongest differential expressed proteins between eMDSCs^high^ and eMDSCs^low^ groups. **(B)** A multispectral IF staining assay showed that the changes of ARID1A expression between eMDSCs^high^ and eMDSCs^low^ groups in 140 primary breast cancer samples from cohort 1. **(C–G)** The ARID1A expression between different groups in **(B)** was analyzed.

We performed a proteomics analysis on mice mammary breast cancer cell line EO771 xenografts collected from SOCS3^KO^ mice (eMDSCs^high^ group) or SOCS3^fl/fl^ mice (eMDSCs^low^ group). Bioinformatic analysis of GO based on the strongest differentially expressed proteins between eMDSCs^high^ and eMDSCs^low^ groups was further employed to conduct functional annotation. Interestingly, the ARID1A-containing BAF chromatin remodeling complexes (mammalian SWI/SNF) ranked among the top 30 of GO enrichment (Figure 4A).

It’s reported that ARID1A depletion accelerates EMT, increases cancer stemness and promotes migration, invasion and angiogenesis in various cancers by reducing the chromatin accessibility of target genes or influencing the post-transcriptional modification process (Wilson et al., 2019; Luo et al., 2020; Wang et al., 2020; Tomihara et al., 2021). We compared the correlation between the number of infiltrated eMDSCs and the expression level of ARID1A protein in 140 primary breast cancer tissues from cohort 1. Consistently, the expression level of ARID1A protein dramatically decreased in eMDSCs^high^ group compared to that in eMDSCs^low^ group using multispectral immunofluorescence IF staining assay (*p* = 0.0021, Figures 4B,C). Further analysis of breast cancer patients with different molecular subtypes revealed that ARID1A expression was significantly lower in eMDSCs^high^ luminal A breast cancer (*p* = 0.0369, Figure 4D), rather than in the luminal B, HER2 and Basal-like subtype (*p* = 0.0917, *p* = 0.4872, *p* = 0.3361, Figures 4E–G). These data are highly consistent with our previous observation that eMDSCs affected the prognosis of patients with luminal A breast cancer. Furthermore, Kaplan-Meier analysis in 140 primary breast cancer tissues from cohort 1 demonstrated that patients with lower ARID1A expression showed a worse outcome (Supplementary Figure S3A), which was reconfirmed by online Kaplan–Meier-Plotter database-analyzed overall survival plot of breast cancer (Supplementary Figure S3B) (Gyorffy et al., 2010). These data suggest that eMDSCs suppress the ARID1A expression to cause poor prognosis in breast cancer patients.

### Early myeloid-derived suppressor cells accelerate epithelial-mesenchymal transition in luminal A breast cancer cells by downregulating ARID1A

**FIGURE 5:**
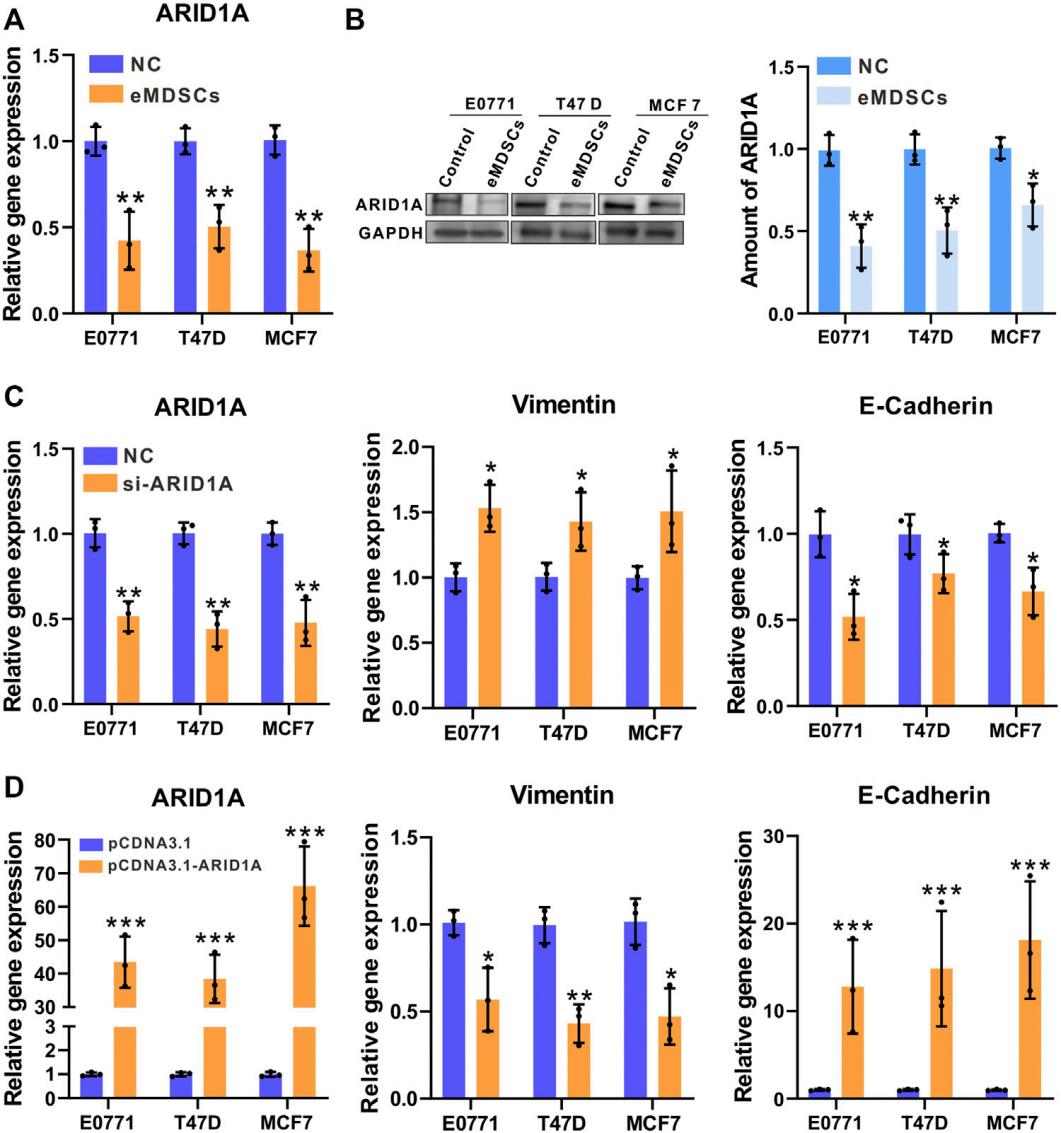
eMDSCs accelerate epithelial-mesenchymal transition in luminal A breast cancer cells by downregulating ARID1A **(A,B)** The mRNA and protein level of ARID1A were determined by RT-PCR and immunoblotting in EO771, T47D and MCF7 cells after cocultured with eMDSCs. **(C)** Quantitative RT-PCR showing expression changes of ARID1A and EMT-related genes in EO771, T47D and MCF7 cells upon ARID1A knocked-down with the transfection of siRNA or vehicle. **(D)** Quantitative RT-PCR showing expression changes of ARID1A and EMT-related genes in EO771, T47D, and MCF7 cells cocultured with eMDSCs upon ARID1A overexpression plasmids or vehicle. *p < 0.05, **p < 0.01, ***p < 0.001.

After co-cultured with eMDSCs, ARID1A showed significant downregulated in both mRNA and protein expression in EO771, MCF-7, and T47D cells, which is consistent with our previous observations in clinical samples (Figures 5A,B). To further explore if downregulation of ARID1A expression is crucial for eMDSCs to promote EMT of luminal A breast cancer cells, we knocked down ARID1A by RNA interference and compared the expression of multiple functional genes by RT-PCR (Figure 5C). As expected, the expression of EMT-related genes was significantly regulated, in which the expression of Vimentin increased but the expression of E-Cadherin decreased (Figure 5C). Subsequently, we established stable EO771, MCF-7, and T47D clones overexpressing ARID1A respectively for further confirming that the expression level of ARID1A is crucial for eMDSCs to promote EMT of luminal A breast cancer cells (Figure 5D). The breast cancer cells transfected with the pCNDA3.1-ARID1A or pCNDA3.1 plasmids were independently cocultured with eMDSCs. Compared to negative control, the E-Cadherin gene increased significantly and Vimentin decreased after ARID1A overexpression (Figure 5D).

**FIGURE 6:**
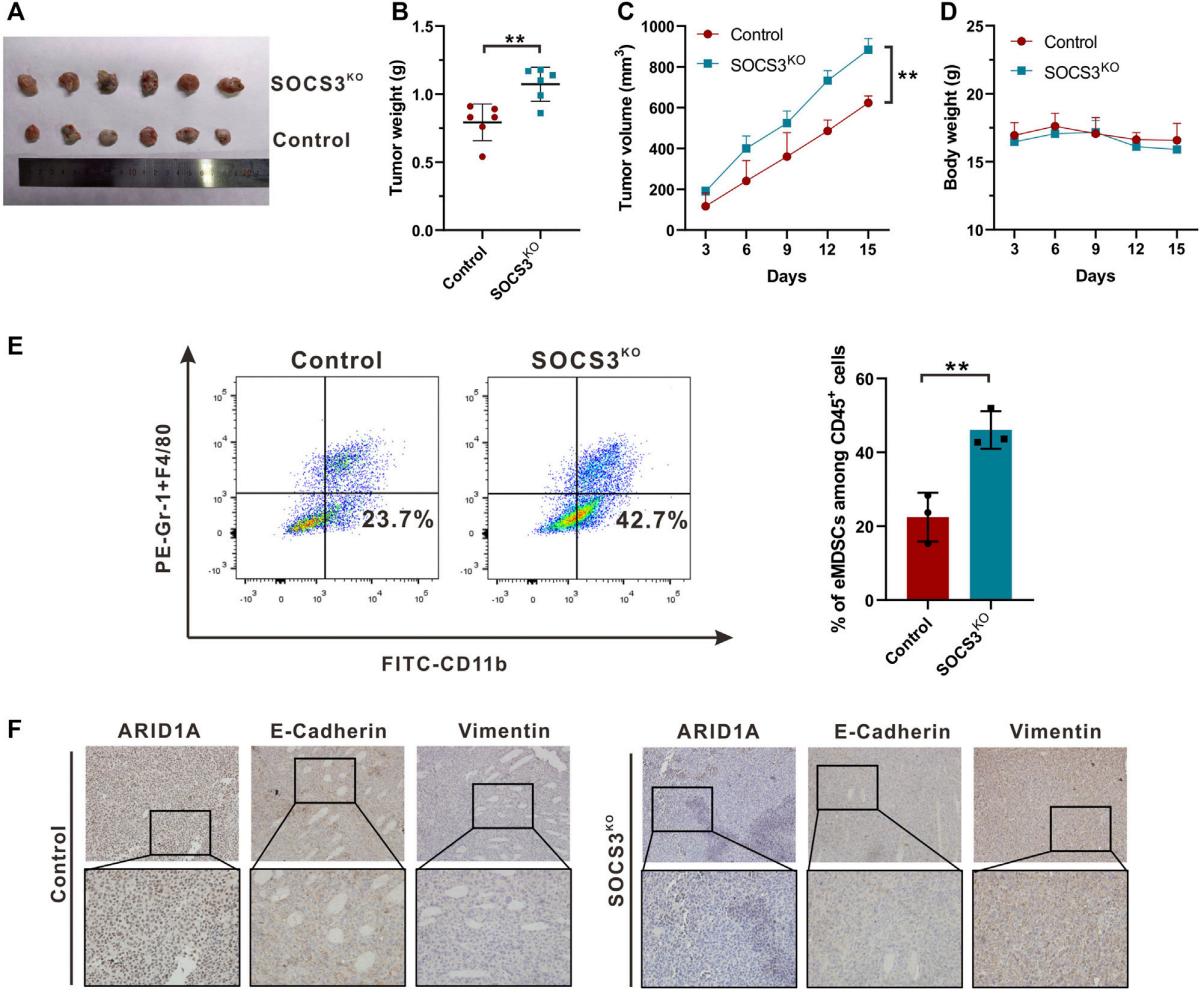
eMDSCs promote tumor growth *via* accelerating EMT process **(A)** Representative images for xenografts model in SOCS3^fl/fl^ and SOCS3^KO^ mice. **(B,C)** The weight and volume of tumors in the SOCS3^KO^ group were significantly greater than those in the control group. **(D)** There was no discernible change in body weight between the SOCS3^KO^ group and the negative control group. **(E)** The percentages of eMDSCs were detected by FCM in primary tumor tissues. **(F)** The images show immunohistochemistry staining for ARID1A, E-Cadherin, and Vimentin in xenografts. *p < 0.05, **p < 0.01.

To assess the impact of eMDSCs on luminal A breast cancer cells *in vivo*, EO771 cells were used to establish subcutaneous xenograft model in SOCS3^fl/fl^ and SOCS3^KO^ mice for further experiments. We found that conditional SOCS3 knockout in myeloid linage strikingly promoted tumor growth (Figure 6A). After 15 days, the weight and volume of tumor in SOCS3^KO^ group were much more than that in control group (Figures 6B,C), whereas there was no discernible change in body weight between the two groups (Figure 6D). Flow cytometry analysis revealed that the number of eMDSCs increased in the tumor tissues of tumor-bearing SOCS3^KO^ mice compared to SOCS3^fl/fl^ mice (Figure 6E; Supplementary Figure S4). Meanwhile, IHC staining showed attenuated levels of ARID1A and E-Cadherin and enhanced expression of Vimentin in highly eMDSCs-infiltrated xenografts (Figure 6F), which suggested that eMDSCs accelerated EMT process and may further promote the initiation of metastasis. Collectively, the above results suggested that eMDSCs accelerated epithelial-mesenchymal transition by downregulating ARID1A in luminal A breast cancer cells.

## Discussion

Growing evidence shows that crosstalk between cancer cells and the surrounding microenvironment appears to plays an important role in the recurrence and metastasis of various cancers (Wang et al., 2018; Hinshaw and Shevde, 2019; Lan et al., 2019; Zavros and Merchant, 2022). MDSCs are a prominent component of the tumor microenvironment and have extraordinary suppressive abilities, including the ability to limit T cell activation, induce NK cell anergy, and influence regulatory T cell accumulation (Ma et al., 2011; Lindau et al., 2013; Hegde et al., 2021). Aside from their impacts on immune responses, MDSCs also contribute to tumor development through a variety of non-immunological pathways, including angiogenesis support, tumor cell stemness promotion, EMT facilitation, and pre-metastatic niche creation (Safarzadeh et al., 2018; Wang et al., 2019; Tang et al., 2021). Since 2013, we have focused on CD33^+^ eMDSCs in human breast cancer tissues, and found they possess potent suppression on T cells proliferation and cytokine production (Yu et al., 2013). We demonstrated that cancer-derived interleukin-6 (IL-6) stimulates STAT3-dependent, nuclear factor-κB-mediated indoleamine 2,3-dioxygenase (IDO) upregulation in eMDSCs which triggers immunosuppressive effects of eMDSCs (Yu et al., 2014). Furthermore, we analyzed the immunosuppressive capacity of eMDSCs in 4T1 mammary tumor-bearing mice and found eMDSCs impaired T cell immunity significantly in SOCS3 deficiency-dependent manner by activating the JAK/STAT signaling pathway (Zhang et al., 2018). However, the mechanism that eMDSCs promote breast cancer recurrence and affect prognosis through EMT has not been fully understood.

In this study, using in-house tissue array and TCGA dataset, we demonstrated that the infiltration of eMDSCs within breast cancer tissues affected the prognosis of luminal A breast cancer patients which was negatively correlated with ARID1A expression *in situ*. Furthermore, we demonstrated that eMDSCs promoted the migration and invasion of luminal A breast cancer cells by accelerating EMT. Finally, we proposed a significant role of eMDSCs in promoting EMT of luminal A breast cancer cells by downregulating ARID1A which caused poor prognosis in luminal A breast cancer patients.

ARID1A is a highly conserved subunit of the BAF complex, which hydrolyzes ATP and uses the resulting energy to mobilize nucleosomes and alter accessibility of chromatin to transcriptional and coregulatory machineries (Clapier et al., 2017). Among the genetic abnormalities reported in ER^+^ breast cancer, mutations are commonly detected in genes encoding the subunits of the BAF chromatin remodeling complexes and ARID1A is the most frequently mutated one in BAF (Garraway and Lander, 2013). ARID1A depletion accelerates epithelial-mesenchymal transition, increases cancer stemness and promotes migration, invasion and angiogenesis in various cancers, including breast cancer, uterus cancer and pancreatic cancer by reducing the chromatin accessibility of target genes or influencing the post-transcriptional modification process (Wilson et al., 2019; Luo et al., 2020; Wang et al., 2020; Tomihara et al., 2021). We found that eMDSCs was negatively correlated with the expression of ARID1A protein and accelerated EMT progression in luminal A breast cancer patients, which suggest a possibility that eMDSCs promote breast cancer cell migration and invasion through down-regulation of ARID1A. The results of cellular experiments verified the authenticity of our idea.

The connection between immune cells and ARID1A had been reported (Shen et al., 2018; Li et al., 2019; Jung et al., 2021), but the regulatory mechanisms have not been fully understood. We found that eMDSCs significantly downregulated ARID1A expression in luminal A breast cancer but the mechanistic events involved in this process are worth more consideration and exploration. It is reported that MDSCs suppress CtBP2 in ovarian cancer cells by inducing miR-101 expression which enhanced cancer cell stemness and spreading potential (Cui et al., 2013). In our previous study, we found that some miRNAs were significantly upregulated in eMDSCs when co-cultured with breast cancer cell line 4T1 (Jiang et al., 2020). To find potential miRNA that may influence the ARID1A expression, we used the miRNA target prediction system “TargetScan” to find high-score potential miRNAs of ARID1A and mmu-miR-342-3p and mmu-miR-9-5p were selected (Supplementary Figure S5A). We validated the expression of the above miRNAs in EO771 cell and found that only mmu-miR-342-3p increased after co-cultured with eMDSCs (Supplementary Figure S5B). Moreover, ARID1A showed significantly downregulated in both mRNA and protein expression after transfected with mmu-miR-342-3p mimics (Supplementary Figure S5C,D). This discovery suggests that eMDSCs downregulated ARID1A expression *via* inducing mmu-miR-342-3p in EO771 cell and miRNA-related post-transcriptional regulation also participated in eMDSCs-induced ARID1A downregulation in luminal A breast cancer.

In summary, our study provided evidence for the tumor promoting function of eMDSCs in breast cancer. We constructed a 21-gene signature in the prediction of the infiltration of eMDSCs within breast cancer tissues. Meanwhile, eMDSCs significantly downregulate ARID1A expression and promote EMT in luminal A breast cancer. These findings shed light on the mechanisms by which eMDSCs could contribute to breast cancer metastasis in the primary tumor microenvironment. The eMDSCs-ARID1A axis was essential for luminal A type of breast cancer metastasis and could be a potential target for metastatic breast cancer therapies.

## Data Availability

The datasets presented in this study can be found in online repositories. The names of the repository/repositories and accession number(s) can be found below: Gene Expression Omnibus, GSE140092.
