# Supplementary material for: Early myeloid-derived suppressor cells accelerate epithelial-mesenchymal transition by downregulating ARID1A in luminal A breast cancer
Source: Front Bioeng Biotechnol. 2022 Oct 18;10:973731. doi: 10.3389/fbioe.2022.973731 (PMC9623091; doi:10.3389/fbioe.2022.973731)
Supplement: Supplementary file 1 [file DataSheet1.pdf]

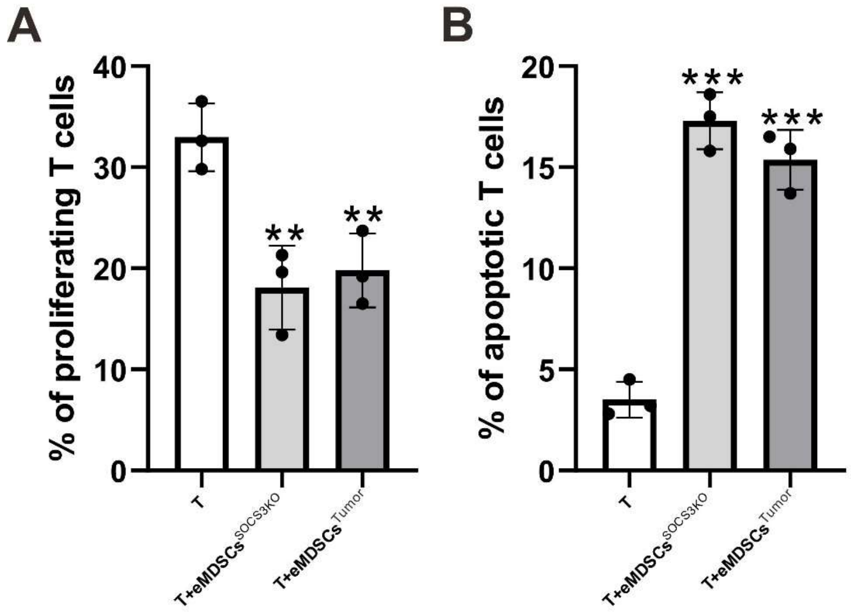

Supplementary Fig. S1. Effects of eMDSCs on T cell proliferation and apoptosis.

T: T cells only; T+eMDSCs^{SOCS3KO}: eMDSCs isolated from the bone marrow of SOCS3^{KO} mice stimulated T cells; T+eMDSCs^{Tumor}: eMDSCs isolated from the tumor tissue of tumor-bearing SOCS3^{KO} mice stimulated T cells.

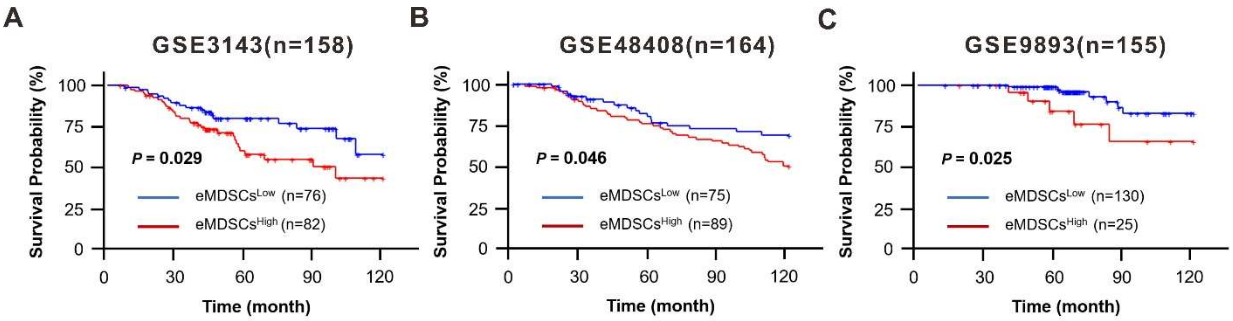

Supplementary Fig. S2. Highly eMDSCs infiltration are significantly correlated with poor prognosis in breast cancer patients

(A-C) Breast cancer samples from different datasets (GSE3143(n=158), GSE48408(n=164), GSE9893(n=155)) were divided into eMDSCs^{high} and eMDSCs^{low} groups using the 21-genes signature, respectively. All results of Kaplan-Meier survival analysis showed that the OS in the eMDSCs^{low} group was longer than the eMDSCs^{high} group.

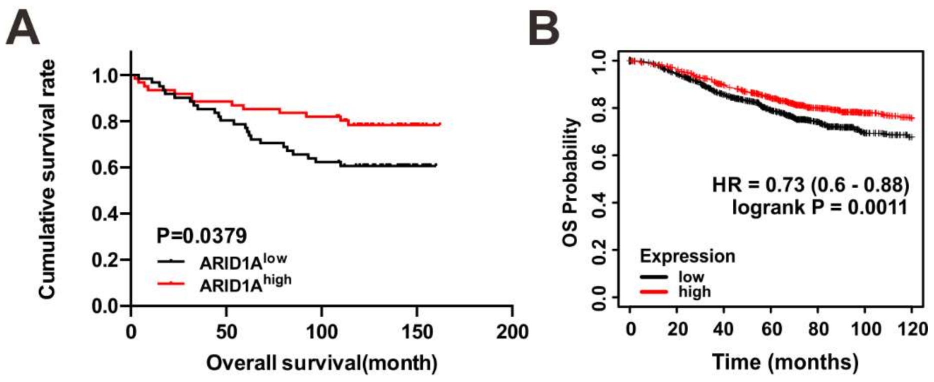

Supplementary Fig. S3. Low expression of ARID1A predicts poor overall survival in breast cancer patients

(A) Kaplan-Meier survival curve showed that lower ARID1A level indicated poor prognosis in 140 cases of primary breast cancer tissues from cohort 1. (B) Kaplan–Meier analysis of OS based on ARID1A mRNA levels using the KM-plotter breast cancer database (http://kmplot.com/analysis). Auto select best cutoff was chosen in the analysis.

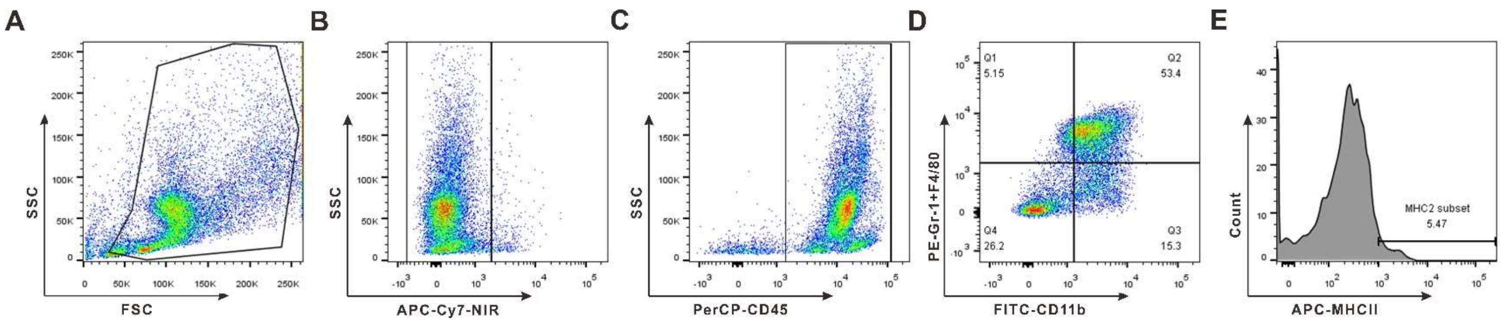

Supplementary Fig. S4. The percentage of eMDSCs in tumor tissues was increased in SOCS3^{KO} mice compared to SOCS3$^{fl/fl}$ mice.

(A-E) eMDSCs were gated using PerCP-labeled anti-CD45, and were stained with APC-Cy7-NIR, FITC-labeled anti-CD11b, PE-labeled anti-Gr-1, PE-labeled anti-F4/80, and APC-labeled anti-MHCII. Cells in Q3 represent the eMDSCs. (1) All cells are gated as parental population according to SSC and FSC features. (2) The NIR negative cells are gated as alive cells. (3) The CD45 positive cells are gated as leukocyte. (4) Both Gr-1 and F4/80 positive cells are regarded as mature neutrophils and monocytes. Therefore, CD11b positive, Gr-1 negative and F4/80 negative cells are gated as eMDSCs(Q3), which express myeloid phenotype but excluding mature neutrophils and monocytes. (5) Because human eMDSCs are HLA-DR low-expressing, we detect the expression of MHCII molecule in mice eMDSCs, which barely express MHCII on surface. Thus, we determine the phenotype of mice eMDSCs as CD11b+Gr-1−F4/80−MHCII−.

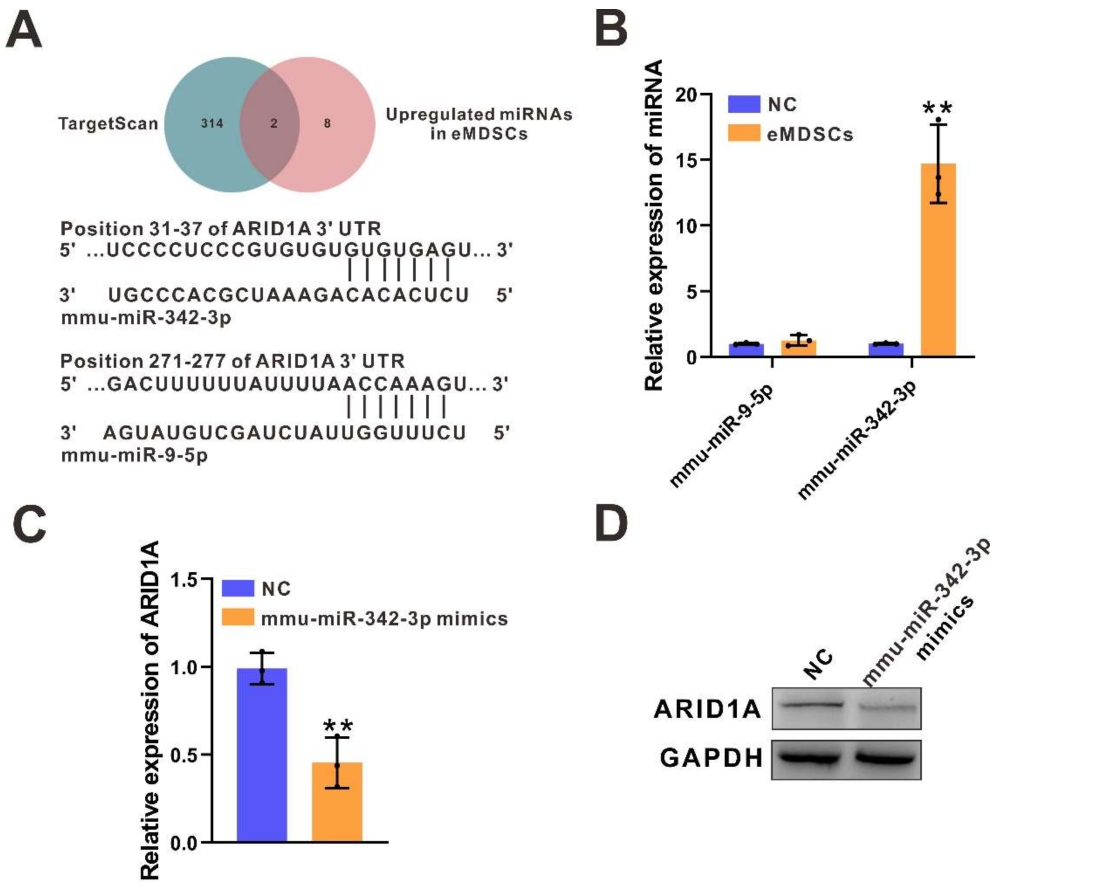

Supplementary Fig. S5. eMDSCs downregulated ARID1A expression via inducing mmu-miR-342-3p in EO771 cell.

(A) The overlap of upregulated miRNAs in eMDSCs and high score potential miRNAs of ARID1A. (B) The expression mmu-miR-342-3p and mmu-miR-9-5p were further confirmed by RT-PCR. (C-D) The mRNA and protein level of ARID1A were determined by RT-PCR and immunoblotting in EO771 after after transfected with mmu-miR-342-3p mimics.
